# Endometrial cells sense and react to tissue damage during infection of the bovine endometrium via interleukin 1

**DOI:** 10.1038/srep07060

**Published:** 2014-11-14

**Authors:** Laura L. Healy, James G. Cronin, I. Martin Sheldon

**Affiliations:** 1 Institute of Life Science, College of Medicine, Swansea University, Singleton Park, Swansea, SA2 8PP, United Kingdom

## Abstract

Cells generate inflammatory responses to bacteria when pattern recognition receptors bind pathogen-associated molecules such as lipopolysaccharide. Cells may also respond to tissue damage by sensing damage-associated molecules. Postpartum bacterial infections of the bovine uterus cause endometritis but the risk of disease is increased by tissue trauma triggered by dystocia. Animals that suffered dystocia had increased concentrations of inflammatory mediators IL-8, IL-1β and IL-1α in vaginal mucus 3 weeks postpartum, but they also had more bacteria than normal animals. *Ex vivo* organ cultures of endometrium, endometrial cells and peripheral blood monocytes did not generate inflammatory responses to prototypical damage molecules, HMGB1 or hyaluronan, or to necrotic cells; although they secreted IL-6 and IL-8 in a concentration-dependent manner when treated with IL-1α. However, necrotic endometrial cells did not accumulate intracellular IL-1α or release IL-1α, except when pre-treated with lipopolysaccharide or bacteria. Endometrial cell inflammatory responses to IL-1α were dependent on the cognate receptor IL-1R1, and the receptor adaptor protein MyD88, and the inflammatory response to IL-1α was independent of the response to lipopolysaccharide. Rather than a typical damage-associated molecule, IL-1α acts to scale the inflammatory response in recognition that there is a combination of pathogen challenge followed by endometrial cell damage.

Innate immunity is an ancient system of defense based on cellular pattern recognition receptors that bind pathogen-associated molecules or molecular patterns (PAMPs) found in prokaryotes but not eukaryotes^1^,^2^. The first functional mammalian pattern recognition receptor to be identified was Toll-like receptor (TLR)4 on macrophages, which binds the lipopolysaccharide (LPS, endotoxin) cell wall component of Gram-negative bacteria^3^. In hematopoietic cells, engagement of TLRs leads to secretion of cytokines such as interleukin (IL)-1β, IL-6, interferon gamma (IFN-γ) and tumor necrosis factor alpha (TNF-α), and chemokines such as IL-8^1^,^2^. Chemokines and cytokines then attract and activate more hematopoietic immune cells to clear the microbes. However, sterile inflammation caused by tissue damage is also typified by production of IL-1, IL-6 and IL-8, and the recruitment of neutrophils and peripheral blood mononuclear cells (PBMCs) to remove cellular debris^4^. The “danger model” proposes that cells use their pattern recognition receptors to sense and respond to alarm signals from injured tissues^4^,^5^. Damage-associated molecules or molecular patterns (DAMPs) are endogenous factors released into the extracellular fluid by dying or necrotic cells, or components of the extracellular matrix^4^,^5^. A prototypical intracellular DAMP is the nuclear protein high molecular group box 1 (HMGB1), which is released by necrotic cells or secreted by activated macrophages, and binds to receptors including TLRs to stimulate inflammation, in a similar manner to the responses to PAMPs^6^,^7^. Hyaluronan is an example of an extracellular matrix DAMP produced during tissue damage, which stimulates inflammation via TLR2 and TLR4^8^. Another mechanism underlying the danger model is the passive release during cell damage of the cytokine IL-1α, which is normally sequestered inside cells^9^. Unlike pro-IL-1β, which has to be cleaved to active IL-1β, pro-IL-1α is biologically active and binds the IL-1R on adjacent cells, leading to secretion of cytokines such as IL-6^9^,^10^. However, there remains uncertainty about the relative contribution of DAMPs and PAMPs to inflammation when there is concurrent tissue damage and infection.

Inflammation is a universal feature of uterine biology after parturition in all mammals associated with tissue damage, and infection of the endometrium mucosal lining of the uterus by bacteria that ascend the female genital tract via the cervix^11^,^12^,^13^. Postpartum involution of the uterus involves remodeling of the extracellular matrix, and repair of damaged endometrium, particularly regeneration of the epithelium that overlies a layer of stromal cells. Elimination of bacteria from the endometrium depends principally on an innate immune response to PAMPs, leading to influx of phagocytic cells such as neutrophils and macrophages. In most species the recovery of the uterus after parturition proceeds without incident but in *Bos taurus* about 40% of dairy cattle develop uterine disease, which causes infertility^11^. The pathogens isolated from the uterus of animals with endometritis include Gram-negative *Escherichia coli*, Gram-positive *Trueperella pyogenes*, and a range of anaerobic bacteria^14^,^15^. Uterine disease is characterized by inflammation of the endometrium, tissue damage and necrosis, and the accumulation of pus in the genital tract^11^,^16^. In addition, endometritis is typified by increased expression of gene transcripts for *IL1A*, *IL1B*, *IL6* and *IL8* in the endometrium^17^,^18^. The cost of treating uterine disease, lost milk production, and replacing infertile animals is about $2 billion per annum for the combined North American and European Union dairy industries^11^. Thus, it is imperative to understand the underlying mechanisms of how cells respond to infection and damage, in order to develop novel approaches for preventing and treating endometritis.

Organ cultures of bovine endometrium respond to *E. coli* and *T. pyogenes* by secreting IL-1β, IL-6 and IL-8^19^. Furthermore, not only hematopoietic cells, but also the epithelial and stromal cells of the endometrium express functional TLR4 for detection of LPS, and TLR1, TLR2 and TLR6 for sensing bacterial lipopeptides; both events leading to secretion of IL-6 and IL-8 *in vitro*^20^,^21^,^22^. Whilst the role of innate immunity in sensing and responding to bacteria via their PAMPs and host cell pattern recognition receptors are well established in the endometrium, bacteria also possess virulence factors that cause tissue damage and necrosis. In particular, all isolates of *T. pyogenes* secrete a cholesterol-dependent cytolysin, Pyolysin, which causes hemolysis, and cytolysis of endometrial cells, especially stromal cells^23^. Physical factors such as dystocia and retained fetal membranes further contribute to endometrial tissue damage and increase the risk of endometritis^24^. However, little is known about how endometrial cells sense and respond to tissue damage and necrosis, whether associated with postpartum physiological changes or enhanced by uterine infection. The present study tested the hypothesis that endometrial cells sense and generate inflammatory responses to DAMPs.

## Results

### Inflammatory mediators are more abundant following dystocia

**Figure 1:**
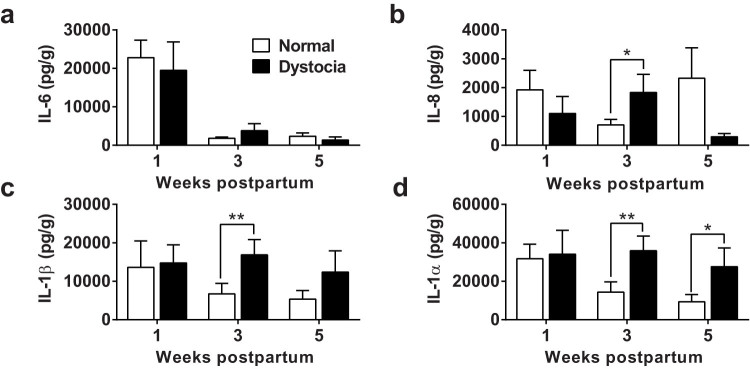
Inflammatory mediator concentrations in postpartum vaginal mucus. Mucus was collected 1, 3, and 5 weeks postpartum, from the vagina of 13 dairy cows that had dystocia (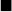) and 39 animals that had a normal parturition (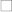). The concentrations of (a) IL-6, (b) IL-8, (c) IL-1β and (d) IL-1α were measured by bovine-specific ELISA and expressed as pg per g mucus. Data are presented as mean + SEM. Data were analyzed by GLM ANOVA, using the Bonferroni post hoc multiple comparison test; values differ between parturition group, ** p < 0.01, * p < 0.05.

To examine whether tissue damage associated with dystocia causes localized inflammation in the postpartum genital tract, vaginal mucus was collected from 52 dairy cows across 5 farms where parturition was scored on a scale of 0 (no intervention, normal parturition) to 3 (severe dystocia attended by a veterinarian). The mucus was processed and the concentrations of inflammatory mediators measured by ELISA. The concentrations of IL-6 were higher in vaginal mucus during the first week than 3 or 5 weeks postpartum, irrespective of whether there had been dystocia or normal parturition (Fig. 1a; p < 0.05). On the other hand, there were higher concentrations of IL-8, IL-1β and IL-1α in the vaginal mucus collected 3 weeks postpartum from 13 animals that had dystocia compared with animals that had normal parturition, and the concentrations of IL-1α remained higher in the vaginal mucus of dystocia animals sampled 5 weeks postpartum (Fig. 1b–d). However, the severity of dystocia was also correlated with the isolation of bacteria in vaginal mucus (r^2^ = 0.99, p < 0.001), and in particular the pathogen *T. pyogenes* was more likely to be isolated from animals that had dystocia than animals with a normal parturition (8/13 vs 8/39, p < 0.05). Thus, the increased genital tract inflammatory responses could be caused by tissue damage and/or bacterial infection *in vivo*.

### Responses to potential DAMPs

**Figure 2:**
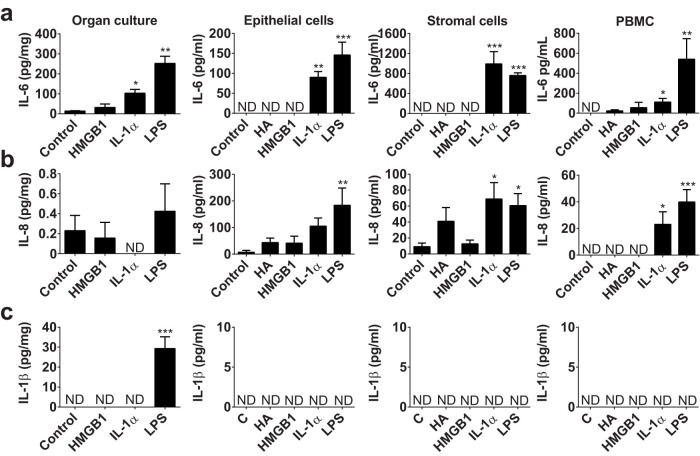
Inflammatory responses to DAMPs. *Ex vivo* organ cultures of endometrium, endometrial epithelial and stromal cells, and peripheral blood mononuclear cells (PBMCs) were cultured for 24 h in control medium, or media containing 10 μg/ml hyaluronan (HA, except organ cultures), 1 μg/ml HMGB1, 10 ng/ml IL-1α, and 0.1 μg/ml LPS. Supernatants were harvested and the accumulation of (a) IL-6, (b) IL-8 and (c) IL-1β measured by ELISA. Data are presented as mean + SEM from 4 independent experiments. Data were analyzed by multivariate GLM, using the Dunnett's pairwise multiple comparison t-test; values differ from control, * p < 0.05, ** p < 0.01, *** p < 0.001, ND = not detected.

To explore whether inflammatory responses seen *in vivo* may relate specifically to tissue damage, we examined endometrial tissue and cellular inflammatory responses to potential DAMPS. *Ex vivo* organ cultures of endometrium, and *in vitro* culture of pure populations of primary endometrial epithelial and stromal cells, and peripheral blood mononuclear cells (PBMCs), were generated^19^,^20^,^21^,^22^,^23^. These were treated with prototypical DAMPs, 1 μg/ml HMGB1, 10 μg/ml hyaluronan (except organ cultures, which already contain abundant hyaluronan), and 10 ng/ml IL-1α, based on concentrations reported in the literature and by the manufacturer^6^,^7^,^8^,^9^. Inflammation was evaluated by measuring IL-6, IL-8, IL-1β, IFNγ and TNFα protein by ELISA. As a positive control, and to gauge the relative intensity of inflammatory responses we used the classical PAMP, 0.1 μg/ml LPS, which stimulates endometrial cell cytokine responses^21^,^22^. As expected, all the culture supernatants accumulated IL-6 and IL-8 in response to LPS (Fig. 2a and b), and only organ cultures accumulated IL-1β in response to LPS (Fig. 2c). However, the supernatants of organ cultures, endometrial cells and PBMCs did not accumulate significantly more IL-6, IL-8 or IL-1β in response to HMGB1 than control (Fig. 2a–c). Similarly, there was no significant accumulation of IL-6, IL-8 or IL-1β in response to the physiological high-molecular-weight polymer of hyaluronan (Fig. 2a–c). Tissue injury also leads to extra-cellular matrix turnover and the accumulation of lower molecular weight fragments of hyaluronan, which may modulate inflammatory responses^8^,^25^. So, epithelial and stromal cells were treated with 10 μg/ml of each of a range of hyaluronan fragments (4–8, 15–40, 75–350 and >950 KDa). However, the different molecular weights of hyaluronan did not stimulate significant accumulation of IL-6 in supernatants from epithelial cells (all values below control; p = 0.99) or stromal cells (all values below the limits of detection of the assay).

The supernatants of endometrial organ cultures, endometrial epithelial and stromal cells, and PBMCs all accumulated IL-6 in response to treatment with IL-1α (Fig. 2a), and endometrial stromal cells and PBMCs secreted IL-8 in response to IL-1α (Fig. 2b); although none of the cultures accumulated IL-1β in response to IL-1α (Fig. 2c). The concentrations of IFNγ and TNFα were below the limits of detection of the assays for all the DAMP and PAMP treatments (<25 pg/ml IFNγ; <5.7 pg/ml TNFα). We conclude that whilst endometrial cells do not respond to the DAMPs, HMGB1 or hyaluronan, the cells respond to IL-1α in a similar manner to their response to a PAMP.

### Cellular production of IL-1α

**Figure 3:**
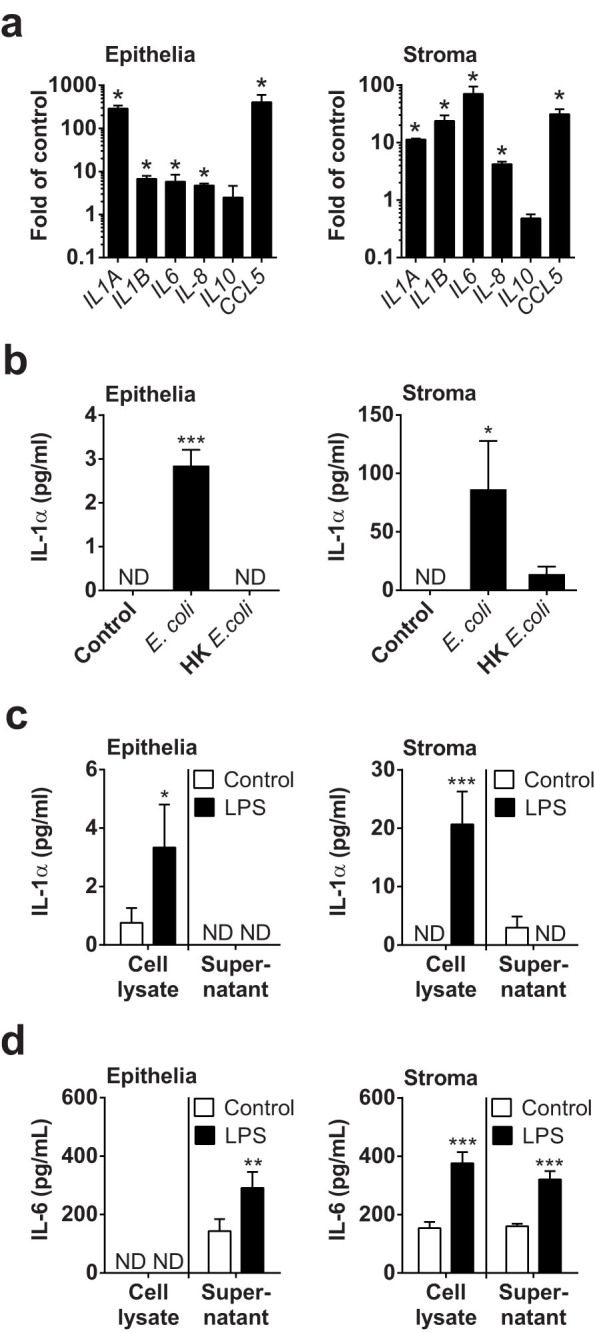
Production of IL-1α by endometrial cells. (a) Endometrial epithelial cells and stromal cells were treated with 0.1 μg/ml LPS for 3 h, and the RNA extracted for cDNA synthesis and qPCR to measure expression of the gene transcripts listed. Data are mean + SEM, expressed as fold of control, from 3 independent experiments. Data were compared with control by Mann Whitney test; values differ from control, * p < 0.05. (b) Endometrial cells were treated with control medium or media containing live *E. coli* or heat-killed (HK) *E. coli* for 24 h and the concentrations of IL-1α measured in cell supernatants by ELISA. Data are mean + SEM from 4 independent experiments. Data were analyzed by GLM ANOVA, using the Dunnett's pairwise multiple comparison t-test; values differ from control, *** p < 0.001, * p < 0.05. (c, d) Epithelial and stromal cells were treated with control medium or medium containing 0.1 μg/ml LPS for 24 h. Cell supernatants were collected, the remaining cells were lysed in an equal volume of lysis buffer, and concentrations of (c) IL-1α and (d) IL-6 measured by ELISA. Data are mean + SEM, from 3 independent experiments. Data were analyzed by GLM ANOVA, using the Dunnett's pairwise multiple comparison t-test; values differ from control, *** p < 0.001, **, p < 0.01, * p < 0.05, ND = not detected.

As vaginal mucus accumulated IL-1α in animals with dystocia and infection (Fig. 1), and the only *in vitro* responses to potential DAMPs were to IL-1α (Fig. 2), the production of IL-1α was explored further in endometrial cells. Treatment of epithelial and stromal cells with 0.1 μg/ml LPS for 3 h increased the expression of *IL1A* gene transcripts (Fig. 3a), as well as mRNA for *IL1B*, *IL6*, *IL8* and *CCL5*, which are genes typically associated with an innate immune response in the endometrium^11^,^17^,^18^. The supernatants of epithelial and stromal cell cultures also accumulated IL-1α protein (Fig. 3b) when challenged with a live strain of our endometrial pathogenic *E. coli*^14^,^26^. However, treatment with heat-killed bacteria, using the same strain of *E. coli*, did not stimulate accumulation of significant amounts of IL-1α in epithelial or stromal cell supernatants (Fig. 3b).

As live but not heat-killed endometrial pathogenic *E. coli* invade and damage cells^14^; and, as IL-1α is normally an intracellular cytokine^10^; we next explored the response of endometrial cells to LPS, measuring the concentration of cytokines in cell lysates as well as in culture supernatants. Treatment with 0.1 μg/ml LPS increased the accumulation of IL-1α protein in lysates of epithelial and stromal cells after 24 h but there was almost no IL-1α detectable in the cell culture supernatants (Fig. 3c). Whereas, IL-6 accumulated in the supernatant of cells that were treated with control medium or with LPS (Fig. 3d). So, IL-1α was principally elaborated following a PAMP stimulus, but the cytokine was retained intracellular in undamaged cells.

**Figure 4:**
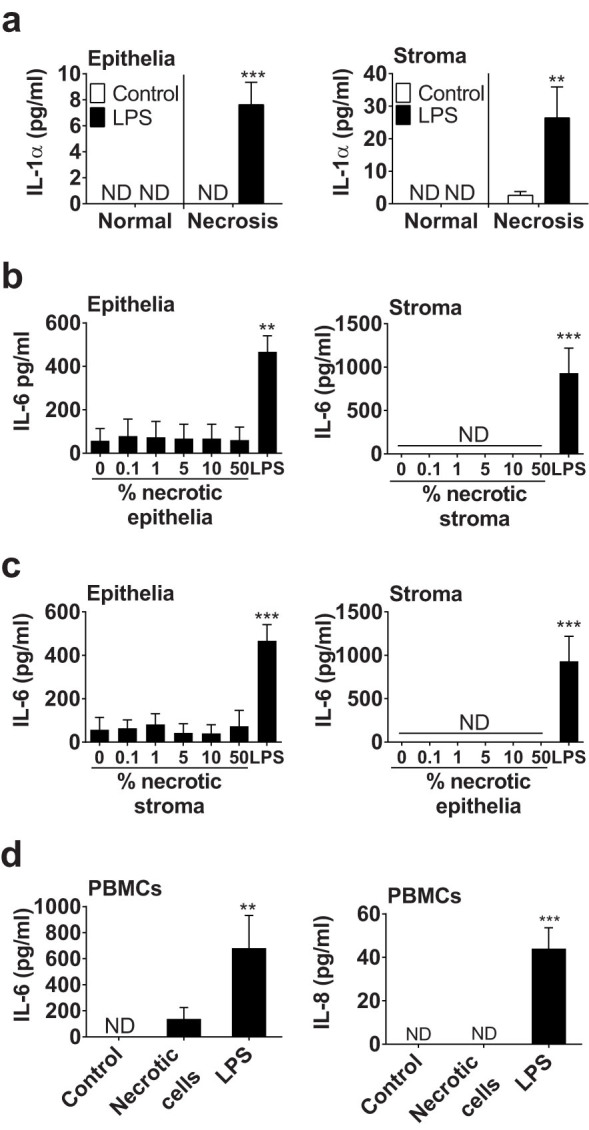
Necrotic cells do not simulate inflammation. (a) Endometrial epithelial and stromal cells were cultured in control medium or medium containing 0.1 μg/ml LPS for 24 h prior to collection of supernatants from normal cells or from cells in which necrosis was induced by cycles of freezing and thawing. Data are mean + SEM, from 3 independent experiments. Data were analyzed by GLM ANOVA, using the Bonferroni post hoc multiple comparison test; values differ from control, *** p < 0.001, ** p < 0.01. (b, c) Endometrial cells were cultured for 24 h in media containing the indicated percent of a solution of necrotic (b) homologous or (c) heterologous cells, with medium containing 0.1 μg/ml LPS used as a positive control. Cell-free supernatants were collected and the concentrations of IL-6 were measured by ELISA. (d) PBMCs were treated with control medium, or media containing 10% necrotic stromal cells or 0.1 μg/ml LPS for 24 h. Cell-free supernatants were collected and IL-6 and IL-8 measured by ELISA. Data are mean + SEM from 4 independent experiments. Data were analyzed by GLM ANOVA, using the Dunnett's pairwise multiple comparison t-test; values differ from control, *** p < 0.001, ** p < 0.01, ND = not detected.

To determine if sterile cell damage alone was sufficient to stimulate the release of IL-1α and induce endometrial cell inflammatory responses, necrotic cells were generated by cycles of freezing in liquid nitrogen and thawing at 37°C, as described previously^27^. However, supernatants of necrotic epithelial or stromal cells only contained significantly more IL-1α compared with normal cells, if the cells were treated with LPS prior to the induction of necrosis (Fig. 4a). The concentrations of IL-1α from necrotic cells in the absence of LPS were only above the limits of detection of the assay for 2 of 7 animals across Fig. 3c and Fig. 4a. However, to determine whether cell-associated IL-1α or other potential DAMPs could stimulate inflammation, endometrial cells were treated with media containing up to 50% of solutions of lysed necrotic cells (Fig. 4b, c). The supernatants of cultured healthy epithelial or stromal cells did not accumulate IL-6 in response to challenge with suspensions of homologous (Fig. 4b) or heterologous (Fig. 4c) necrotic cells. Similarly, a 10% suspension of necrotic cells did not significantly increase the accumulation of IL-8 above control from epithelial (18.9 ± 8.9 vs. 5.3 ± 5.3 pg/ml IL-8) or stromal cells (11.6 ± 4.2 vs. 9.2 ± 4.4 pg/ml IL-8). Furthermore, treatment of PBMCs with 10% necrotic stromal cells did not stimulate significant increases in IL-6 or IL-8 (Fig. 4d). The concentrations of IL-1β in supernatants of endometrial cells and PBMCs treated with suspensions of necrotic cells were below the limits of detection of the ELISA. Taken together, these data provide evidence that IL-1α was not a typical DAMP associated with sterile cell damage in the bovine endometrium, and sterile necrotic cells did not provoke endometrial cell inflammatory responses. Rather, IL-1α accumulated when there was a combination of pathogen challenge followed by cell damage.

### Impact of IL-1α on endometrial cells

**Figure 5:**
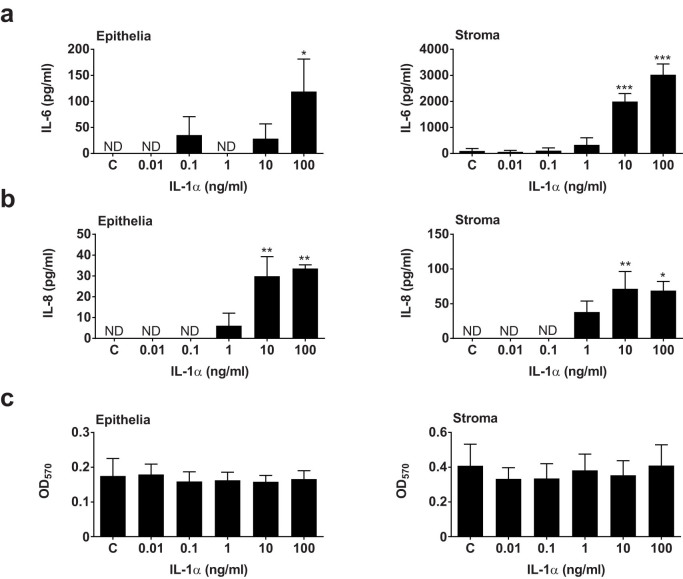
Endometrial cellular responses to IL-1α. Endometrial epithelial and stromal cells were treated for 24 h with control medium or media containing the indicated concentrations of IL-1α. Supernatants were collected for the measurement of (a) IL-6 and (b) IL-8 by ELISA, and (c) cell viability was examined by MTT assay. Data are presented as mean + SEM of 4 independent experiments. Data were analyzed by GLM ANOVA, using the Dunnett's pairwise multiple comparison t-test; values differ from control, *** p < 0.001, ** p < 0.01, * p < 0.05, ND = not detected.

To further explore the effect of IL-1α on endometrial cells, epithelial and stromal cells were treated with a range of concentrations of IL-1α from 0.01 to 100 ng/ml. Treatment with IL-1α stimulated both epithelia cells and stromal cells to secrete more IL-6 (Fig. 5a) and more IL-8 (Fig. 5b), without significantly affecting cell survival (Fig. 5c). Interestingly, IL-1α did not induce the production of IL-1β, with concentrations below the limits of detection of the ELISA. We conclude that endometrial cells mount concentration-dependent IL-6 and IL-8 inflammatory responses to IL-1α.

### Endometrial cells express function IL-1R1

**Figure 6:**
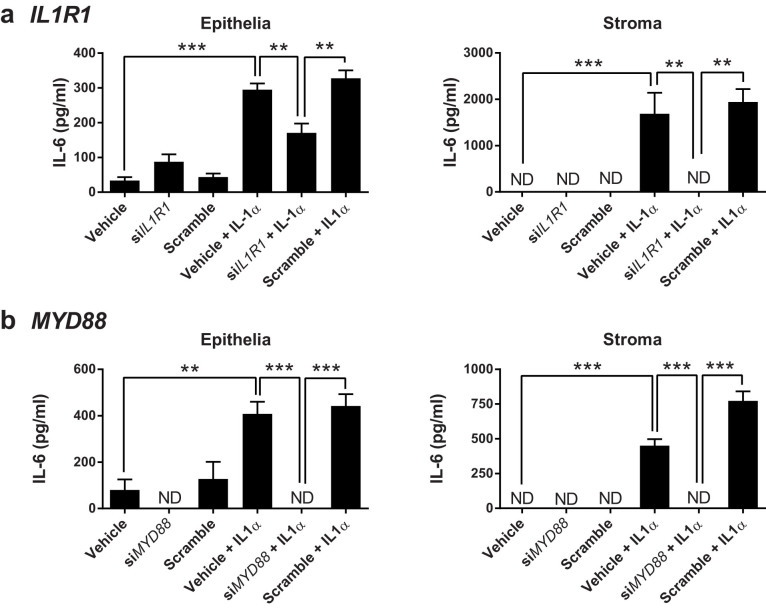
IL-1α acts via the IL-1R and MyD88. Endometrial epithelial and stromal cells were cultured in medium with vehicle or transfected with siRNA targeting (a) *IL1R1* or (b) *MYD88*, or with a scramble siRNA sequence, followed by treatment with control medium or medium containing 10 ng/ml IL-1α for 24 h. Supernatants were collected and the concentrations of IL-6 measured by ELISA. Data are presented as mean + SEM of 4 independent experiments. Data were analyzed by GLM ANOVA, using the Bonferroni post hoc test; values differ, *** p < 0.001, ** p < 0.01, ND = not detected.

As endometrial cells responded to IL-1α, we examined the functionality of the cognate receptor IL-1R1^10^, and the MyD88 adapter protein required for IL-1R1 signaling, using measurement of IL-6 in stromal cell supernatants as a marker of cellular responses to IL-1α. There was reduced accumulation of IL-6 in response to 24 h treatment with 10 ng/ml IL-1α in epithelial cells and in stromal cells that had been transfected with siRNA targeting *IL1R1* (Fig. 6a) or *MYD88* (Fig. 6b), compared with cells transfected with a scramble siRNA; and, there was no significant effect of the siRNA on cell survival, as determined by MTT assay (all OD_570_ values within ±10% of control). Thus, endometrial cells generate their response to IL-1α via the cytokine's cognate receptor, IL-1R1.

### Responses to IL-1α and LPS are independent

**Figure 7:**
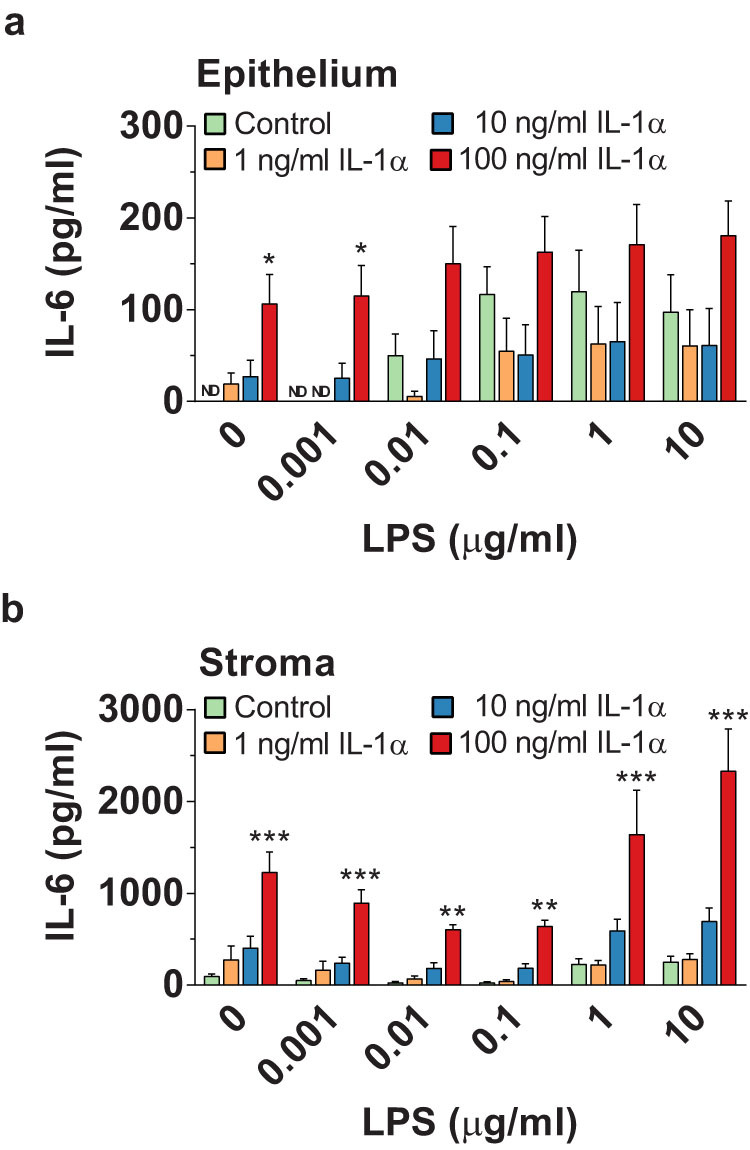
Independent impact of IL-1α and LPS. Endometrial epithelial cells (a) and stromal cells (b) were treated for 24 h with control medium or media containing both IL-1α and LPS using the range of IL-1α and LPS concentrations indicated. Supernatants were collected and the concentrations of IL-6 measured by ELISA. Data are presented as mean + SEM of 4 independent experiments. Data were analyzed by GLM ANOVA, using the Bonferroni post hoc test; values differ from control, within LPS-concentration group, *** p < 0.001, ** p < 0.01, * p < 0.05; ND = not detected.

Having established that the release of IL-1α depends on pathogen challenge in combination with cell damage, and that endometrial cells respond to IL-1α as well as LPS, our final question was whether cellular responses to IL-1α and LPS were synergistic? Endometrial cells were simultaneously treated with a range of concentrations of IL-1α and LPS, and the accumulation of IL-6 measured by ELISA. There was a significant effect on the accumulation of IL-6 from epithelial cells of IL-1α treatment (Fig. 7a, p < 0.001) but not LPS treatment (p = 0.17), and the interaction of IL-1α and LPS was not significant (p = 0.99). For stromal cells, there was a significant effect on IL-6 secretion of IL-1α treatment (Fig. 7b, p < 0.001) and LPS treatment (p < 0.001), but the interaction of IL-1α and LPS treatments was not significant (p = 0.99). These data indicated that cellular responses to IL-1α and LPS are independent.

## Discussion

**Figure 8:**
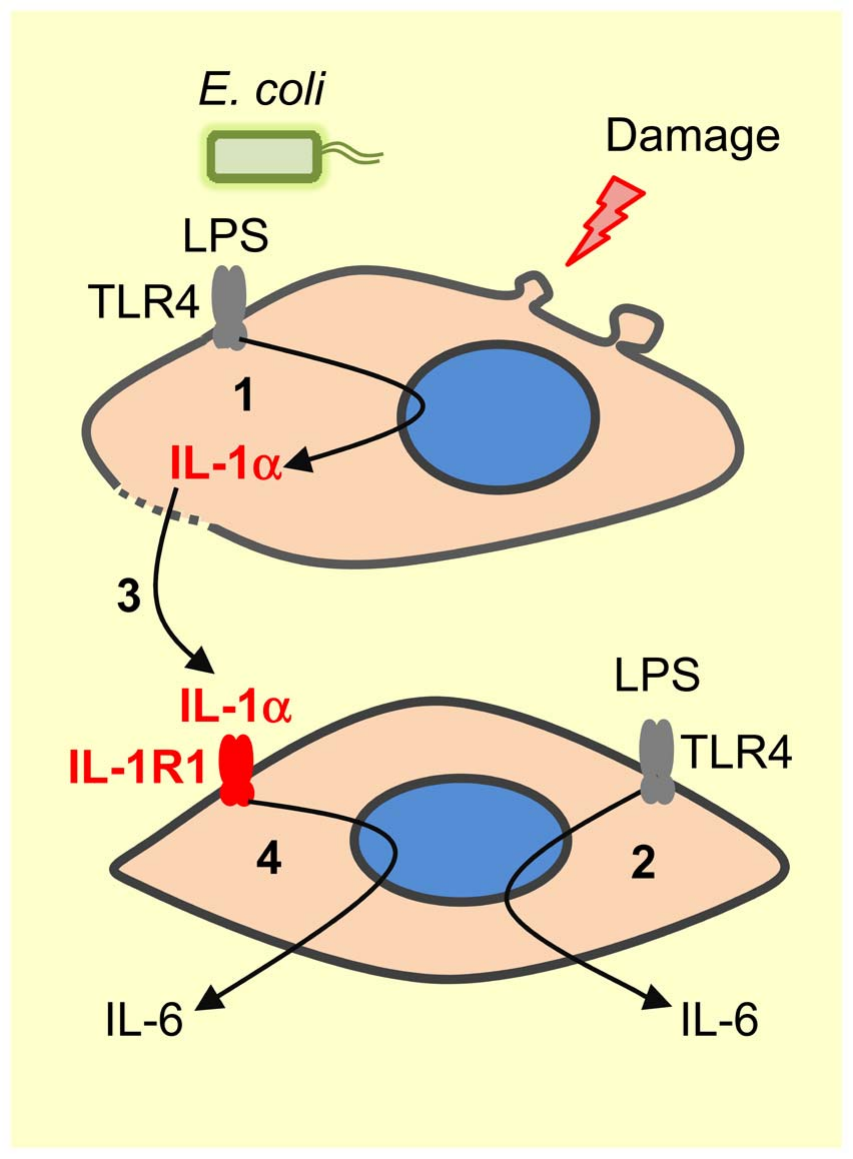
IL-1α acts as a factor to increase inflammation during infection and damage of cells. Model showing that activation of TLRs following the binding of PAMPs, such as LPS from *E. coli*, stimulates accumulation of intracellular IL-1α (1), as well as secretion of other inflammatory mediators such as IL-6 (2). Subsequently, damage of cells releases IL-1α into the extracellular fluid (3), where it binds IL-1R1 on nearby cells to stimulate further secretion of IL-6 (4), and augment cellular inflammatory responses to pathogens when there is cell damage.

The differentiation of “self” versus “non-self” is a fundamental concept in immunity. From files to mammals, pattern recognition receptors, such as TLRs, sense PAMPs to detect microbes^1^,^28^. However, when it comes to damaged “self” the mechanisms are less well defined and harder to rationalize. The danger model is predicated on chemical alarm signals, such as DAMPs, being released from cells and tissues damaged by trauma, pathogens or toxins; and then DAMPs alert nearby healthy cells to the problem^5^. A wide range of endogenous components of cells and extracellular matrix have been proposed as DAMPs, and many are thought to bind to pattern recognition receptors^4^,^29^. Typical DAMPs released by necrotic cells and tissues include HMGB1, hyaluronan and IL-1α^6^,^7^,^8^,^9^. Unfortunately life is more complicated and, like many inflammatory conditions, postpartum endometritis is associated with both tissue damage and bacterial infection^11^. Endometrial cells sense and generate inflammatory responses to bacteria via pattern recognition receptors, including TLR4 for LPS, and TLR2 for bacterial lipopeptides^21^,^22^. However, as the impact of tissue damage on endometritis was unclear, in the present study we explored the role of DAMPs. As expected, we found that dystocia and infection are rarely independent *in vivo*, although the localized accumulation of IL-1α in vaginal mucus of diseased animals was notable. *In vitro*, endometrial tissue or cells did not generate inflammatory responses to necrotic cells or the prototypical DAMPs hyaluronan or HMGB1. However, cells generated inflammatory responses to IL-1α, although IL-1α was only released when there was a pathogen stimulus followed by cell damage. The epithelial and stromal cell inflammatory responses to IL-1α required the cognate receptor IL-1R1 and adapter protein MyD88, and cellular responses to IL-1α and LPS were independent, so that IL-1α increased the response above that to LPS alone. We conclude that rather than a typical DAMP, IL-1α acts to scale the endometrial cell inflammatory response in recognition that there is a combination of pathogen challenge followed by host cell damage (Fig. 8).

*Bos taurus* provided a useful model to investigate infection and tissue damage because the bovine endometrium is always infected with bacteria after parturition^15^,^30^, and the risk of endometritis is increased by dystocia or other causes of tissue trauma such as retained fetal membranes or the presence of *T. pyogenes*^16^,^24^,^31^. In the present study, dystocia was associated with a greater localized inflammatory response, as characterized by the accumulation of more IL-1β, IL-8 and IL-1α in vaginal mucus 3 weeks postpartum compared with healthy animals. These inflammatory responses at the protein level in the vaginal mucus reflect previous findings of more abundant gene transcripts for the same inflammatory mediators in the postpartum endometrium^17^,^18^,^32^. Furthermore, these responses are typical of innate immunity in mammals in general^1^,^33^; and of responses in the bovine endometrium in particular^21^,^22^. To specifically examine the role of DAMPs, we used HMGB1, hyaluronan, IL-1α and necrotic endometrial cells. Following tissue damage, extracellular matrix hyaluronan is bound by TLR4 or TLR2, and intracellular HMGB1 by TLR9^6^,^7^,^8^. So, we anticipated detecting inflammatory responses to hyaluronan and HMGB1 because endometrial cells express TLR2, TLR4 and TLR9^17^,^34^. However, we were surprised to find that there was no significant stimulus of IL-6, IL-8, IL-1β, IFNγ or TNFα release from the endometrium or cells in response to hyaluronan, HMGB1 or necrotic cells. It is possible that other mediators might be produced; for example, IL-12 is secreted by dendritic cells treated with necrotic cell^27^. Alternatively, necrotic cells might stimulate other effects such as enhancing epithelial cell survival^8^. Mature IL-1β was detectable in postpartum vaginal mucus and in organ cultures treated with LPS, although IL-1β was not detected following treatment of endometrial or peripheral blood cells with LPS. In future studies, it would be interesting to explore which cells produce IL-1β in the endometrium, and macrophages, Natural Killer cells, monocytes, and neutrophils are likely candidates. One consideration is that release of mature IL-1β requires formation of the multi-protein inflammasome^9^,^35^. In the present study, IL-1α stimulated the production of IL-6 and IL-8 from endometrial cells, and the cellular responses were dependent on the concentration of IL-1α. Whilst inflammatory responses to IL-1α have not been reported previously for bovine endometrial cells, IL-1α also stimulates the endocrine function of endometrial cells, including increased secretion of prostaglandin F_2α_ and E_2_^36^,^37^.

The principal receptor for IL-1α is IL-1R1^10^,^38^. The endometrial cellular responses to IL-1α in the present study required IL-1R1 and the receptor's adapter protein MyD88, as determined by the reduced IL-6 secretion in response to IL-1α for cells transfected with siRNA targeting *IL1R1 or MYD88*. The present findings support studies in mice where animals lacking IL-1R1 or MyD88, but not deficient in TLR2, TLR4 or TLR9, had reduced inflammatory responses to tissue injury^38^. Furthermore, as in our study, the response required non-hematopoietic cells and IL-1α was the key to the inflammatory response, whilst HMGB1 was regarded as unimportant^38^. Of course during infection and tissue damage, host cells are exposed to both PAMPs and DAMPs, and it was notable that the quantitative response to 10 ng/ml IL-1α and 0.1 μg/ml LPS were similar. Furthermore, the inflammatory responses to LPS and IL-1α were independent, which likely reflects binding to their respective receptors, TLR4 and IL-1R1. This independence of response may present a therapeutic opportunity, by exploiting pharmacological inhibitors of the response to IL-1α without affecting TLRs^39^.

As IL-1α was the only evident potential DAMP in our system, we explored the production of IL-1α. The accumulation of IL-1α in the vaginal mucus of animals with dystocia and infection 3 and 5 weeks but not 1 week postpartum was intriguing because if IL-1α was a DAMP, one might expect the highest concentration soon after the trauma of dystocia. Furthermore, cellular necrosis did not lead to accumulation of intracellular or extracellular IL-1α, whereas *E. coli* or LPS stimulated the production of IL-1α. Furthermore, intracellular IL-1α was only released when there was cell damage, which supports previous observations^9^,^10^. Interestingly, the 33 KDa precursor form of pro-IL-1α is biologically active as well as 17 KDa mature IL-1α, which distinguishes it from pro-IL-1β that must be cleaved to mature IL-1β to act biologically^10^. As pro-IL-1α is active it is often considered as an alarmin for sterile tissue damage^9^,^39^. However, in our system IL-1α only stimulated more inflammation when there was endometrial cell damage as well as infection (Fig. 8). The mechanisms underlying the release of IL-1α requires more investigation. One potential mechanism is that some cells use the intracellular decoy receptor IL-1R2 to bind IL-1α and prevent activity, even in necrotic cells^40^; although the IL-1α may be released if there is also infection. It is notable that the postpartum endometrium expresses abundant *IL1R2* and *IL1A* mRNA, particularly in animals that develop endometritis^17^. Whilst the present study provides several lines of evidence for the role of IL-1α in endometrial cell responses to infection followed by cell damage, we cannot exclude the possibility that IL-1β may also be involved. Scaling of the immune response is an important concept because many microbes possess PAMPs that bind TLRs, yet not all these microbes cause pathology or warrant a florid inflammatory response by the host^41^. Factors such as cellular necrosis, and toxins such as cholesterol-dependent cytolysins, are features of microbes that cause pathology, and so likely require more active responses from the host. It would be interesting to explore whether the *in vivo* inflammation we detected during the postpartum period involves responses to other factors that scale the inflammatory response. For example, prokaryotic DNA from live bacteria may be important to alert the immune system to an active infection^42^. Alternatively, other forms of cell death and damage may stimulate inflammation^35^. Indeed, the *T. pyogenes* cholesterol-dependent cytolysin pyolysin causes endometrial cell lysis and hemolysis, although endometrial cells do not secrete inflammatory mediators in response to pyolysin-induced cytolysis^23^.

In conclusion, the present study examined the role of tissue damage in the induction of inflammation in endometrial cells. Surprisingly, prototypical DAMPs or necrotic cells were not important. In addition, whilst intracellular IL-1α provoked cellular inflammatory responses, rather than behaving like a classical DAMP, IL-1α acted only when there was both infection and damage. So, IL-1α might best be considered as a factor that further stimulates innate immunity when there is host cell damage in addition to bacterial infection.

## Methods

### Collection of samples from animals

To examine whether dystocia was associated with localized inflammation in the female genital tract, vaginal mucus was sampled from 52 cows, across 5 farms, at 1 week, 3 weeks, and 5 weeks postpartum. Farm staff recorded whether calving was normal or whether there was dystocia requiring manual intervention, using a score from 0 (normal) to 3 (severe dystocia). Each herd was visited weekly by a veterinarian, and one, three and five weeks postpartum, mucus was collected from the vagina using a clean, lubricated, gloved hand inserted through the vulva and into the vagina, and the mucus was stored at −20°C. The mucus was processed prior to measurement of inflammatory mediators by ELISA as described previously^43^. Briefly, 2 g mucus was placed in 10 ml of cytolyt solution (40% Methanol: 60% distilled water) and mixed with 0.1% Dithiothreitol (Sigma-Aldrich) until the mucus was disrupted, followed by centrifugation at 3000 × g for 15 min, and collection of the supernatant.

### Ethical approval

All experiments were performed in accordance with the approved guidelines and regulations, and the ethical approval of the UK Government Home Office (license number PPL 40/3478).

### Organ and cell culture

Uteri with no gross evidence of genital disease or microbial infection were collected from cattle processed as part of the normal work of an abattoir, as described previously^19^,^21^,^22^. *Ex vivo* organ cultures of endometrium were generated as described previously, using 8 mm diameter biopsy punches^19^. Endometrial epithelial and stromal cell populations were isolated, and the absence of immune cell contamination confirmed by FACS analysis, as described previously^21^,^22^. Organ cultures were placed in 12-well plates (TPP, Trasadingen, UK) in 2 ml/well culture medium (RPMI-1640 medium, 50 IU/ml of penicillin, 50 μg/ml of streptomycin and 2.5 μg/ml of amphotericin B, Sigma; with 10% fetal bovine serum, Biosera, East Sussex, UK), and epithelial and stromal cells were plated at 1.5 × 10^5^ cells/ml in 1 ml/well culture medium in 24-well plates to examine cytokine or chemokine responses, or processed in 6-well plates for siRNA experiments, as described below. Tissue and cells were incubated at 37°C in a humidified atmosphere of air with 5% CO_2_. Peripheral blood mononuclear cells (PBMCs) were isolated as described previously^23^; plated at 2 × 10^3^ cells/ml in culture medium in 24-well plates, and treated 24 h later.

### Endometrial tissue and cell responses to DAMPs

Endometrial organ cultures, endometrial epithelial and stromal cells and PBMCs were treated for 24 h with 1 μg/ml HMGB1 (R&D, Abingdon, UK), 10 ng/ml IL-1α (2B2, Upper Heyford, UK), or 10 μg/ml hyaluronan (R&D), based on concentrations reported in the literature and by the manufacturer^6^,^7^,^8^,^9^; with 0.1 μg/ml ultrapure LPS from *E. coli* (Invivogen, Toulouse, France) used as a positive control. In independent experiments endometrial cell responses were examined using: 10 μg/ml of a range of four different molecular weight hyaluronan (4–8, 15–40, 75–350 and >950 KDa; R&D); a range of concentrations of IL-1α (0.001 to 10 ng/ml); and, a range of concentrations of a suspension of necrotic epithelial or stromal cells (0.1 to 50%), generated by 5 cycles of freezing in liquid nitrogen followed by thawing at 37°C, as described previously^43^. To test for synergistic cellular responses, endometrial cells were concurrently treated for 24 h with medium containing IL-1α (0, 1, 10 or 100 ng/ml) and LPS (0, 0.001, 0.01, 0.1, 1 and 10 μg/ml). Supernatants were collected and stored at −20°C. The number of cells was evaluated by MTT assays, as described previously^23^. Experiments were repeated on at least 3 independent occasions, with treatments applied to duplicate replicate wells.

### Accumulation of IL-1α

Expression of endometrial cell *IL1A* mRNA was evaluated by qPCR following 3 h treatment with control medium or 0.1 μg/ml LPS. Production of IL-1α protein was examined by treating endometrial cells for 24 h with antibiotic-free control medium or medium containing 0.1 μg/ml LPS, or 1 × 10^8^ CFU/ml live endometrial pathogenic *E. coli*^26^, or an equivalent number of the same *E. coli* heat-killed, as described previously^23^. Supernatants were collected and stored at −20°C, whilst the cells were either subjected to freeze/thaw necrosis^43^, or washed in PBS and lysed in 1 ml/well lysis buffer (PBS, 2% v/v Triton X-100, 1% w/v SDS); and, cell-free supernatants were collected following centrifugation at 14,000 × g for 10 min at 10°C.

### siRNA

Primary endometrial epithelial and stromal cells were transfected with Lipofectamine™ RNAiMAX Reagent (Invitrogen, Paisley, UK) and siRNA conducted exactly as described previously^21^,^22^. The siRNA duplexes were designed using Dharmacon's siDESIGN Center (Thermo Fisher Scientific, UK) targeting *IL1R1* (sense 5' CAAGAAUACAGAAGGAAUAUU 3'; antisense 3' UUGUUCUUAUGUCUUCCUUAU 5'; from NCBI Reference Sequence NM_001206735), and targeting *MYD88* as described previously^21^.

### ELISA

Concentrations of IL-1β, IL-6, IFNγ, TNFα and IL-8 in culture supernatants were measured by ELISA according to manufacturer's instructions (Bovine IL-1β Screening Set ESS0027, Bovine IL-6 Screening Set ESS0029, Thermo Scientific, Cramlington, UK; Bovine IFNγ DuoSet DY2300, Bovine TNFα DuoSet DY2279, Human CXCL8/IL-8 DuoSet DY208, all R&D). The IL-8 assay has previously been shown to cross-react with bovine IL-8^44^. The inter-assay and intra-assay coefficients of variation were all <10%; the limits of detection were 12.5 pg/ml for IL-1β, 75.0 pg/ml for IL-6, 25 pg/ml for IFNγ, 83.2 pg/ml for TNFα, and 5.7 pg/ml for IL-8.

To measure bovine IL-1α, an IL-1α sandwich ELISA was developed using 96-well microtitre ELISA plates (Greiner Bio-one; 675061; Stonehouse, UK) coated with 2 μg/ml polyclonal rabbit anti-bovine IL-1α capture antibody (PB0331B, Kingfisher Biotech, USA) diluted in carbonate/bicarbonate buffer (0.2 M Na_2_CO_3_, 0.2 M NaHCO_3_, pH 9.4; C3041; Sigma) for 18 h at room temperature. The plates were washed three times with PBS 0.05% Tween 20 (Sigma), and blocked with PBS 4% fish-skin gelatin for 1 h. A 7-point standard curve was generated using recombinant bovine IL-1α (800 pg/ml to 12.5 pg/ml; RP0097B; Kingfisher Biotech, Inc.), and test samples loaded in duplicate wells, prior to incubation for 1.5 h at room temperature. The plates were then washed and 0.2 μg/ml biotinylated polyclonal rabbit anti-bovine IL-1α (PBB0332B; Kingfisher) added for 2 h, before the plates were washed again, and the reaction visualized using 3,3′,5,5′-tetramethylbenzidine (BD Biosciences, Oxford, UK) for 15 min. The reaction was stopped using 0.5 M sulphuric acid, and absorbance measured at 450 nm using a plate reader (Fluostar, BMG Labtech, Ortenbug, Germany), with IL-1α concentrations calculated by four parameter logistic regression. The upper and lower limits of detection were 800 pg/ml and 2.6 pg/ml, respectively. Cross-reactivity was <1% for bovine IL-10 and CCL2 (Kingfisher Biotech), IL-6 and IL-1β (Thermo Scientific), and interferonγ and TNFα (R&D). The intra-assay coefficient of variation for 200, 400, or 800 pg/ml IL-1α was 3.6, 6.9 and 8.1%, respectively, and the inter-assay coefficient of variation was 6.6, 5.9 and 8.2%, respectively.

### Quantitative PCR (qPCR)

Extraction of cellular RNA, cDNA synthesis, and quantitative PCR were performed using the primers and procedures described previously^17^,^45^.

### Statistical analysis

Data are presented as arithmetic mean and SEM. Statistical analyses were performed using SPSS 20.0 (SPSS Inc, Chicago, IL, USA) with the animal as the designated statistical unit, and p < 0.05 considered significant. Differences in proportions were examined using Chi-square test, and correlations were examined by Pearson Square. Comparisons were made between treatments using GLM multivariate ANOVA, and Bonferroni or Dunnett's post hoc multiple comparison tests, except for qPCR data where the Mann-Whitney test for non-parametric data was used.

## Author Contributions

L.L.H. conducted most of the experiments, prepared figures and completed data analysis. J.G.C. developed the bovine IL-1α ELISA and assisted with siRNA experiments. I.M.S. conceived the project and wrote the manuscript. All authors contributed to experimental design and commented on the manuscript.
